# Has Data Quality of an Antimicrobial Resistance Surveillance System in a Province of Nepal Improved between 2019 and 2022?

**DOI:** 10.3390/tropicalmed8080399

**Published:** 2023-08-04

**Authors:** Sweety Upadhaya, Jyoti Acharya, Maria Zolfo, Divya Nair, Mahesh Kharel, Anjana Shrestha, Basudha Shrestha, Surendra Kumar Madhup, Bijendra Raj Raghubanshi, Hari Prasad Kattel, Piyush Rajbhandari, Parmananda Bhandari, Subhash Thakur, Gyani Singh, Lilee Shrestha, Runa Jha

**Affiliations:** 1 National Public Health Laboratory, Kathmandu 44600, Nepal; jyotigan30@gmail.com (J.A.); mrmaheshkharel@gmail.com (M.K.); anjananshup@gmail.com (A.S.); shresthalilee@gmail.com (L.S.); runa75jha@gmail.com (R.J.); 2 Institute of Tropical Medicine, 2000 Antwerp, Belgium; mzolfo@itg.be; 3 International Union against Tuberculosis and Lung Diseases, 75001 Paris, France; divya.nair@theunion.org; 4 Kathmandu Model Hospital, Kathmandu 44600, Nepal; basudha111@gmail.com; 5 Dhulikhel Hospital, Dhulikhel 45200, Nepal; sur2036@hotmail.com; 6 KIST Medical College Teaching Hospital, Lalitpur 44700, Nepal; raghu2_47@yahoo.co.in; 7 Tribhuwan University Teaching Hospital, Kathmandu 44600, Nepal; hariprasadkattel@gmail.com; 8 Patan Hospital, Patan Academy of Health Sciences, Lalitpur 44700, Nepal; piyushrajbhandari@pahs.edu.np; 9 Sukraraj Tropical and Infectious Disease Hospital, Kathmandu 44600, Nepal; parmananda_bhandari@yahoo.com; 10 Paropakar Maternity and Women’s Hospital, Kathmandu 44600, Nepal; subasmt5@gmail.com; 11 Kanti Children’s Hospital, Kathmandu 44600, Nepal; gyanisingh365@gmail.com

**Keywords:** Global Antimicrobial Resistance and Use Surveillance System (GLASS), antimicrobial resistance, data quality, National Public Health Laboratory, SORT IT, operational research, South-East Asia, LMIC

## Abstract

An operational research study was conducted in 2019 to assess the quality of data submitted by antimicrobial resistance (AMR) surveillance sites in the Bagmati Province of Nepal to the National Public Health Laboratory for Global Antimicrobial Resistance and Use Surveillance System (GLASS). Measures were implemented to enhance the quality of AMR surveillance by strengthening capacity, improving infrastructure, implementing data sharing guidelines, and supervision. The current study examined reports submitted by surveillance sites in the same province in 2022 to assess whether the data quality had improved since 2019. The availability of infrastructure at the sites was assessed. Of the nine surveillance sites in the province, seven submitted reports in 2022 versus five in 2019. Completeness in reporting improved significantly from 19% in 2019 to 100% in 2022 (*p* < 0.001). Timely reports were received from two sites in 2019 and only one site in 2022. Specimen–pathogen consistency in accordance with the GLASS guidelines for urine, feces, and genital swab specimens improved, with ≥90% consistency at all sites. Overall, the pathogen–antibacterial consistency improved significantly for each GLASS priority pathogen. The study highlights the importance of dedicated infrastructure and institutional arrangements for AMR surveillance. Similar assessments covering all provinces of the country can provide a more complete country-wide picture.

## 1. Introduction

Globally, antimicrobial resistance (AMR) is a major political, social, and economic problem. The burden of AMR is continually increasing in lower- and middle-income countries (LMIC), and Nepal is no exception [1]. The Global Action Plan on AMR, introduced by the World Health Organization (WHO) in 2015, has provided guidance to countries on tackling AMR, with microbiological surveillance as a key action [2]. Following this, the WHO created the Global Antimicrobial Resistance and Use Surveillance System (GLASS), a platform to track AMR across the globe, enabling international comparison, analysis, and the sharing of AMR data. Countries are invited to report aggregated data on the frequency of AMR among high-priority pathogens once a year to the GLASS platform. The ability of such a platform to generate actionable data is contingent on the submission of high-quality data from participating countries in a timely manner, which requires the availability of infrastructure in terms of trained human resources and equipment at the AMR surveillance sites. These are some of the bottlenecks at the surveillance sites, particularly in resource-limited laboratory settings [3].

Nepal has been reporting to GLASS since 2017. In 2019–2020, an operational research (OR) study (hereafter referred to as the “first OR study”) was conducted to assess the quality of AMR surveillance data sent to the National Public Health Laboratory (NPHL), the National Reference Laboratory for AMR surveillance that receives, aggregates, and submits national data to GLASS [4]. In this first OR study, nine AMR surveillance sites from Bagmati Province (formerly Province 3) in Nepal were investigated. Only five of these sites submitted AMR surveillance reports to NPHL during the study period. A considerable volume of data was reported from these sites, with some deficiencies in the quality of data: consistency with the GLASS standards ranged from 77 to 92%, while the completeness of reporting varied from 88 to 100% across the sites. Reports were often not submitted to NPHL in a timely manner. The study also found that four “non-reporting” surveillance sites in the same province lacked dedicated personnel for data entry and basic facilities such as a computer or Internet connectivity [4]. The first OR study was conducted as part of the Structured Operational Research and Training IniTiative (SORT-IT) coordinated by the WHO-TDR Special Program for Research and Training in Tropical Diseases [5].

Around the same time as the first OR study (2019–2020), discussions between key stakeholders and external development partners (Fleming Fund Country Grant for Nepal-FFCGN and WHO) in the “Human Health AMR Surveillance Technical Working Group” (HH-AMR TWG) were initiated. These discussions were supported with findings of the OR study that enabled: (i) the procurement of computers and Internet connectivity for AMR surveillance sites; (ii) the development of a country-specific AMR surveillance protocol that included GLASS criteria for “specimen–pathogen” and “pathogen–antibacterial” combinations; (iii) the training of data personnel at AMR surveillance sites. Simultaneously, NPHL initiated discussions with software developers with support from FFCGN to explore data management options for uniformity in reporting.

The two OR studies were conducted within the routine AMR surveillance program using the same data quality indicators but at two different time points. Thus, they provided an opportunity to assess whether the various activities being conducted to strengthen AMR surveillance in Nepal had resulted in improved data quality.

The aim of the current study (hereafter referred to as the “second OR study”) was to assess if there had been any change in the quality of data received at NPHL for GLASS reporting since the first OR study, and if the availability of infrastructure for surveillance at selected sites in Bagmati Province had improved.

## 2. Materials and Methods

### 2.1. Study Design

The study consisted of a comparison of quality indicators of routinely collected AMR surveillance data, measured through cross-sectional assessments conducted during 2019–2020 (first OR study) and 2022 (second OR study).

### 2.2. Setting

#### 2.2.1. General Setting

Nepal is a small developing country in Asia with seven provinces and a total population of 30 million. It is a land-locked country bordered by India in the south and China in the north. There are 26 AMR surveillance sites covering all the provinces in the country, among which Bagmati Province (formerly Province 3) has the highest number of surveillance sites (ten sites). This province has a population density of 300 per square kilometers and accounts for 20% of the country’s population [6]. Therefore, this province was chosen for assessment in the first and second OR studies.

#### 2.2.2. Specific Setting

##### Process of AMR Surveillance and Reporting to the GLASS Platform in Nepal

Hospital-based laboratories participate as surveillance sites in AMR surveillance and are expected to submit AMR data monthly to the NPHL. These laboratories serve populations ranging from 40,000 to 4 million, and mostly receive community-based samples. The sites submit culture and sensitivity reports for a variety of samples (e.g., blood, urine, feces, genital swabs, etc.) to the NPHL. The number of records submitted per site in their monthly reports to NPHL ranged between 1378 and 26,133 from July to December 2022.

The surveillance sites submit monthly reports to the NPHL, listing the results of all specimens that underwent antibacterial susceptibility tests (AST) in their laboratories each month. The sites may use different data collection systems (paper-based methods or electronic forms with the use of different software) to generate their monthly reports. The reports are shared with the NPHL via email communication. At the NPHL, the data in these reports are cleaned, collated, and duplicates are removed by using Open Data XLS Transformer (or ODX), which can be mapped to automatically cleanse and standardize spreadsheets ahead of loading them into an analytics platform. The reports are analyzed as per GLASS requirements using the Clinical and Laboratory Standards Institute (CLSI) guidelines. Results of the AST for eight priority human bacterial pathogens [*Escherichia coli* (*E. coli*), *Klebsiella pneumoniae* (*K. pneumoniae*), *Acinetobacter baumannii* (*A. baumannii*), *Staphylococcus aureus* (*S. aureus*), *Streptococcus pneumoniae* (*S. pneumoniae*), *Salmonella* species., *Shigella* species, and *Neisseria gonorrhoeae* (*N. gonorrhoeae*)] isolated from four clinical specimens (blood, urine, feces, and genital swabs) are submitted to the GLASS platform annually [7].

The NPHL regularly supervises the laboratories by making periodic monitoring visits, although not all sites may be visited in a given year. The NPHL further ensures the quality of laboratories by sending regular External Quality Assessment Scheme samples (following the NPHL EQAS SOP) every three months, followed by feedback on their performance.

At the time of the first OR study (2019–2020), 21 surveillance sites reported to NPHL, but the data from only 14 sites were complete enough to be uploaded to the GLASS platform. Currently, 25 surveillance sites are submitting data to the NPHL, with the data of 23 sites included in the last 2021 GLASS report.

##### Steps Taken to Improve AMR Surveillance in Nepal

**Figure 1:**
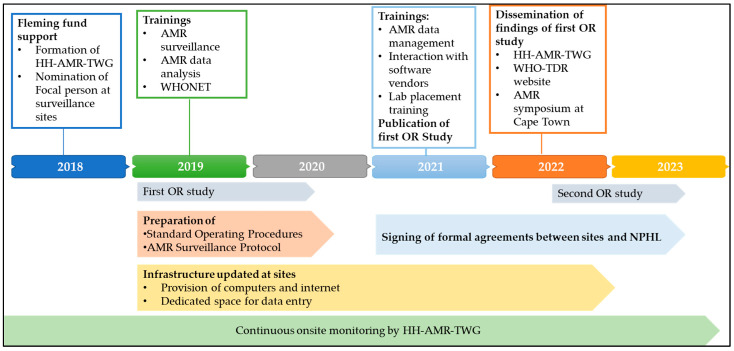
Steps taken to improve the surveillance of antimicrobial resistance in Nepal between 2018 and 2022. Abbreviations: AMR: antimicrobial resistance; WHONET: free software developed by the WHO collaborating center for AMR surveillance; WHO: World Health Organization; OR: operational research; NPHL: National Public Health Laboratory; HH-AMR TWG: Human Health AMR Surveillance Technical Working Group, which includes representatives from the Ministry of Health and Population, Ministry of Agriculture and Livestock Development, Department of Environment, Department of Health Services, Department of Drug Administration, Central Department of Microbiology, surveillance site personnel, WHO, and the Fleming fund country grant for Nepal.

Since 2018, concerted efforts have been made to improve the quality of the AMR data reported to NPHL. As mentioned in the Introduction, the AMR national program has taken certain steps to improve the quality of the AMR data reported to the NPHL. This has included the development of standard operating procedures, the AMR surveillance protocol [8], formalization of the terms of reference for surveillance sites, and the training of data entry operators and staff at surveillance sites (Figure 1).

### 2.3. Study Period for Current Study

The study period was from July to December 2022.

### 2.4. Study Sites and Inclusion Criteria

The Bagmati Province in Nepal has ten AMR surveillance sites, of which nine are human health surveillance sites and these were included in the study. The animal health surveillance site in the province was excluded as it does not report to GLASS. The nine sites were coded as Sites A–I to maintain confidentiality.

At the time of the first OR study (2019–2020), five (Sites A, B, C, D, and E) of these nine sites were sending regular data to the NPHL and were considered as “reporting” sites, while the four sites (Sites F, G, H, and I) that had not submitted data for three consecutive months prior were considered as “non-reporting” sites. Currently, seven of the nine sites are “reporting sites”, while the other two (Sites H and I) continue to be “non-reporting” sites.

In this study, the detailed laboratory records of the positive bacterial culture of AMR pathogens (referred hereafter as “positive records”) from the reports sent to the NPHL for the months July–December 2022 by the seven “reporting” surveillance sites were included.

Sites F, G, H, and I, which were “non-reporting” surveillance sites in the first OR study, were visited and assessed for dedicated human resources, AMR training, WHONET (free software developed by WHO collaborating center for AMR surveillance [9]) training, data analysis training, number and area of rooms dedicated to data entry, computer, Internet service, agreement with the NPHL, and institutional restrictions on data sharing.

### 2.5. Variables, Sources of Data, and Data Collection

All of the reporting sites included in this study submitted their monthly reports to the NPHL in the form of MS Excel spreadsheets. The positive records in the monthly reports submitted were assessed for the quality of data. The quality of reporting was assessed in terms of completeness, timeliness of reporting, “specimen–pathogen consistency”, and “pathogen–antibacterial consistency”.

For “pathogen–antibacterial consistency”, the results of AST for the GLASS priority pathogens (*E. coli*, *K. pneumoniae*, *A. baumannii*, *S. aureus S. pneumoniae*, *Salmonella* spp., *Shigella* spp., and *N. gonorrhoeae*) were extracted from the submitted reports.

A structured questionnaire was used to assess the availability of basic infrastructure and specific requirements in the four previously “non-reporting” AMR surveillance sites. The principal investigator from the NPHL filled in the structured questionnaire by visiting these sites and interacting with the microbiology laboratory personnel involved in AMR surveillance activities. The availability of the physical infrastructure was noted based on the observations made by the investigator.

### 2.6. Data Entry, Analysis, and Statistics

All positive records in the monthly reports submitted by the surveillance sites to the NPHL were extracted in the form of an MS Excel spreadsheet (Microsoft Corporation, 2018) and analyzed using STATA^®^ (version 16.0 Copyright 1985–2019).

Completeness: A record was considered complete if data were available for all of the fields that are mandatory for GLASS reporting as follows: age, gender, origin (inpatient/outpatient), date of specimen collection, specimen, isolate, and antibacterial susceptibility results. The percentage of complete records out of all of the records examined was calculated. Completeness for each site was compared between the two OR studies using the two-proportion Z-test. A *p*-value less than 0.05 was considered significant.

Timeliness of reporting to the NPHL: The deadline for the submission of reports by the site to the NPHL is the last working day of the following month. For each site, the percentage of reports that were submitted within the deadline out of the total reports received during the study period was calculated. For the reports that were not submitted within the deadline, a delay in submission was calculated as the number of days between the date of receipt of the report at the NPHL and the deadline for that month. The delay was summarized as the median (interquartile range) number of days for each site.

The proportion of reports that were submitted in a timely fashion at each site and the median delay in submission was presented for the period of the first OR study (January–June 2019) and the current study (July–December 2022) for each site.

Specimen–pathogen consistency: Specimen–pathogen consistency was reported as the percentage of records in which the specimen–pathogen combination was consistent with the GLASS [7] criteria out of the total number of positive records. This was reported cumulatively, and for each site for each of the four specimen types (blood, urine, feces, and genital swabs).

Pathogen–antibacterial consistency: The pathogen–antibacterial combination in each positive record was checked for consistency with the GLASS [7] criteria. A record was considered consistent only if the antibacterials for which susceptibility testing was reported were in accordance with the GLASS criteria for the pathogen reported in the record. Pathogen–antibacterial consistency was reported as the percentage of consistent reports out of the total number of positive records for each site.

Specimen–pathogen consistency and pathogen–antimicrobial consistency for each site were compared between the two OR studies using the two-proportion Z-test.

Availability of infrastructure: The current status of infrastructure and specific requirements at the four sites that were found to be non-reporting in the first OR study (two of which have now become “reporting sites”) were described. Basic infrastructure refers to the basic facilities and equipment required by the AMR surveillance site to send the AMR reports to the NPHL. Specific requirements include requirements (other than the basic infrastructure) such as the availability of a data entry person; training received on AMR surveillance, data management, and WHONET; agreement with the NPHL and institutional restrictions.

## 3. Results

In this study, the number of reporting sites increased from five to seven. In the first OR study (2019–2020), 1584 positive records from five reporting sites were screened for consistency in specimen–pathogen and pathogen–antibacterial combination, as per the GLASS criteria. In the current study, conducted in 2022 (second OR study), a total of 5302 positive records from seven reporting sites were screened. A comparison of the data quality between the two OR studies was conducted in the “reporting” sites. The availability of infrastructure was assessed in the four sites that were “non-reporting” during the first OR study.

The results of the completeness of reporting, timeliness of reporting, specimen–pathogen consistency, pathogen–antibacterial consistency, and availability of infrastructure are presented below. For each of these, the results are organized as: (1) a comparison of the findings between the first and second OR studies, and (2) a description of the findings for the two sites that have recently begun reporting to the NPHL.

### 3.1. Completeness of Reporting of AMR Surveillance Data

There was a significant improvement in the completeness of reporting during the second OR study compared to the first OR study. All mandatory fields (age, gender, origin, date of specimen collection, specimen, isolate and antibacterial susceptibility results) in the AMR surveillance report were completed in each record by all sites (Table 1).

### 3.2. Timeliness of Submission of AMR Surveillance Reports to NPHL

While two sites (Sites C and E) had submitted all reports without any delay as per the first OR study, only one site submitted all reports (Site E) without any delay during the current study.

In the newly reporting Sites F and G, two of the six expected AMR surveillance reports from Site F and one from Site G were received within the deadline of submission at the NPHL. The median delay in the submission of reports was 31 days and 117 days for sites F and G, respectively (Table 2).

### 3.3. Specimen-Pathogen Consistency

#### 3.3.1. Comparison of Specimen–Pathogen Consistency between the First and Second OR Study

Consistency in the specimen–pathogen combination according to GLASS in reports received from AMR surveillance sites is shown in Table 2. The consistency in specimen–pathogen combination improved at all sites for urine, feces, and genital swabs. However, this consistency declined significantly for blood specimens at two sites (Sites B and E).

#### 3.3.2. Specimen–Pathogen Consistency in Newly Reporting Sites

Among the two sites that were “non-reporting” sites in the previous study and who had started reporting to the NPHL only recently, the specimen–pathogen consistency in blood was 60% and 90%, respectively. Site G showed 100% consistency in urine and genital swabs (Table 3).

### 3.4. Pathogen–Antibacterial Consistency

#### 3.4.1. Comparison of Pathogen–Antibacterial Consistency between the First and Second OR Study

**Figure 2:**
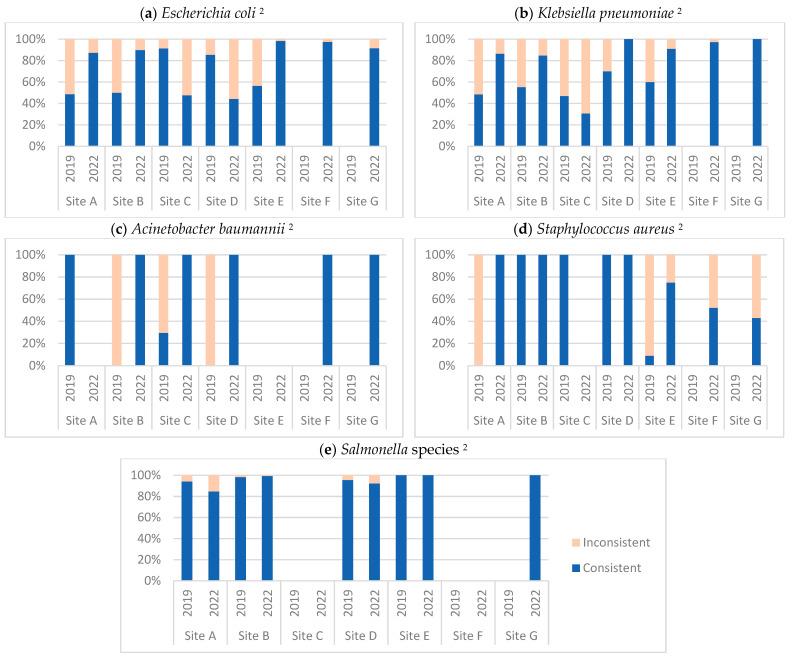
Consistency in the pathogen–antibacterial combination of the AMR surveillance data according to GLASS from five AMR surveillance sites in Bagmati Province, Nepal, during July–December 2022 compared to January–June 2019 ^1^. ^1^ Data for the period January–June 2019 has been published and can be accessed at https://doi.org/10.3390/tropicalmed6020060 (last accessed on 1 August 2023). ^2^ In each graph, the *y*-axis represents the percentage of records where the pathogen–antibacterial combination was consistent with the GLASS guidelines out of all records that reported the pathogen. Sites F and G had not submitted any data during January–June 2019.

Consistency in the pathogen–antibacterial combination of AMR surveillance data according to GLASS from the reporting sites for five pathogens (*E. coli*, *K. pneumoniae*, *A. baumannii*, *S. aureus*, and *Salmonella* spp.) is shown in Figure 2. The exact numbers and proportions of consistent records and tests of statistical significance are provided in Supplementary Table S1. Overall, there was a significant improvement in consistency for all pathogens when data from all sites were compiled together. However, there were some inconsistencies at the individual site level. For the *E. coli* isolates, significant improvement in consistency was observed at Sites A, B, and E, while there was a significant decline at Sites C and D. For the *K. pneumoniae* isolates, significant improvement in consistency was observed at all sites except Site C. For the *A. baumannii* and *S. aureus* isolates, significant improvement in consistency was observed at all sites. For the *Salmonella* species, significant improvement in consistency was observed at Site D. For the *Shigella* and *N. gonorrhoeae* isolates that were reported only from Site B, there was zero consistency in the previous assessment, but the consistency improved to 100% and 80%, respectively (Table S1).

#### 3.4.2. Pathogen–Antibacterial Consistency in Newly Reporting Sites

The pathogen–antibacterial consistency was close to 100% for all bacteria at the newly reporting sites (F and G) except for *S. aureus*, for which only 43% of records were consistent (Figure 2).

### 3.5. Availability of Basic Infrastructure and Specific Requirements

Two sites (Sites F and G) that were non-reporting in the first OR study started reporting consistently to the NPHL since 2022. While these sites had all the basic infrastructure required for reporting in place, the number of microbiology staff decreased at all sites. Dedicated data entry rooms with computers and Internet connectivity were now available at all, except at one site (Site I). At all sites where an informal verbal agreement on data sharing with the NPHL existed previously, a formal signed agreement was now in place. Therefore, none of the sites had any restrictions on data sharing (Table 4).

Two sites (Sites H and I) continued to remain non-reporting sites in the current study. Site H had all the basic infrastructure required for reporting in place. However, Site I did not have a dedicated room, computers, or Internet connectivity for data entry (Table 4).

## 4. Discussion

This OR study was conducted to assess whether there had been any change in the quality of AMR surveillance data received at the NPHL for GLASS reporting, and if the availability of the infrastructure for surveillance at selected sites in Bagmati Province had improved since a previous OR study conducted in 2019. Two of the nine sites in the province that were “non-reporting sites” in 2019 had started reporting to the NPHL regularly since 2022 after the provision of computers and Internet service at one site, and training to the data personnel at the other site. Overall, the quality of reporting in terms of consistency in the specimen–pathogen and pathogen–antibacterial combination, and completeness had improved. Delay in the submission of reports to the NPHL continued to be an issue.

The study’s strengths are that all records in the reports submitted during a six-month period in Bagmati Province were assessed, thus minimizing the selection bias. The study adhered to GLASS standards for assessing the data quality, which are accepted and followed globally. The study was conducted and reported in accordance with the STROBE (Strengthening the Reporting of Observational Studies in Epidemiology) guidelines [10].

A limitation of this study is that it was conducted in only one province, with a limited number of sites, so the findings cannot be generalized for the whole country. Since the interventions to improve AMR surveillance were implemented throughout the country, we were not able to include a control group, and hence it was not possible to assess whether the observed improvements could be attributed to these interventions alone.

This study offers key insights into the current status of the reporting of AMR surveillance data in Bagmati Province. First, two sites in the province that were “non-reporting” sites at the time of the first OR study are now “reporting” sites. At one of these sites, reporting was enabled by the provision of computers and Internet service, which was not available at the time of the first study [4]. At the other site, personnel for data entry had been identified and had then been trained in AMR surveillance and data management. Two surveillance sites continued to remain “non-reporting” sites. These sites were not supported by the FFCGN for infrastructure strengthening. One of them did not have dedicated data entry personnel and a computer, while another had institutional restrictions on data sharing. For the latter, though a formal agreement with the NPHL came into effect in October 2022, the site faced challenges in extracting data from their laboratory information system, but it is expected to start reporting soon. These findings emphasize the need for adequate infrastructure and institutional arrangements for AMR surveillance, as has been stated in other studies from the South-East Asia region and LMICs [11].

Second, the completeness of reporting had improved significantly from 19% to 100%. All seven mandatory GLASS reporting fields were being completed by all sites. The following measures taken by the AMR surveillance program to improve data management at the sites could have facilitated this. (i) All sites used electronic data entry platforms that limit skipping mandatory fields during entry. This is an advantage over the paper-based formats that were used previously [12]. (ii) Data management and reporting as per the AMR surveillance protocol were included in the training package and all sites had been trained. (iii) Interactions with software vendors resulted in the harmonization of different software that were used by the sites so that there was uniformity in reporting as per the GLASS requirements. (iv) The NPHL started using software to extract data as per the GLASS requirements from reports submitted by the surveillance sites. The software is able to flag missing fields and implausible values in the reporting fields, based on which the NPHL team provides timely feedback to the sites [13]. (v) The NPHL team provides continuous support to the sites with regular follow-up for the submission of reports, data quality, and troubleshooting. (vi) Formal agreements for data sharing with institutional heads have helped in enhancing commitment and accountability toward reporting at sites.

Third, although the number of sites reporting to the NPHL had increased, the number of timely reports received per site had reduced between the two studies. Only one site was able to submit all reports on time and submission was delayed by three to 66 days. The reasons for the delay in the submission of reports at each site could be the reduction in the number of dedicated data entry personnel, the non-availability of IT support, slow speed of their Internet connection, or a lack of computers dedicated to data entry. The same bottlenecks have been reported in a previous OR study [4].

Fourth, the data quality in terms of specimen–pathogen consistency and pathogen–antibacterial consistency had improved, but there is room for further improvement. Specimen–pathogen consistency in the blood specimens had declined by 75% and 25% at two sites. At the two sites that had recently begun reporting to the NPHL, specimen–pathogen consistency in blood was 60% and 90%, respectively. This was largely because the reporting of commensals like Coagulase Negative Staphylococcus (CONS) was considered inconsistent as per the GLASS guidelines. However, tertiary-care sites might be justified in reporting CONS as a pathogen in specific situations [14,15,16]. This is an area that needs further investigation with an in-depth review of hospital records for inconsistent reports, and checks to assess whether these sites follow any site-specific reporting protocols that may differ from the GLASS guidelines.

The pathogen–antibacterial combination was consistent with the GLASS guidelines at all except two sites for the *E. coli* isolates. At both of these sites, it was noticed that the AST for one of the mandatory antibacterial agents for a few *E. coli* isolates were not being analyzed. Both of these sites were performing AST in accordance with GLASS for the other isolates. Therefore, the reason for inconsistency in the *E. coli* isolates could be a shortage of the specific antibacterial discs for performing AST.

To summarize, it is encouraging to note that the multi-pronged efforts of the AMR surveillance program have resulted in the improved quality of AMR data in all aspects except timeliness. Timeliness is critical as prescription practices and treatment guidelines are informed by the most current antibacterial susceptibility patterns. Timely reports can also detect sudden outbreaks of multi-drug resistant pathogens and help in early AMR containment [11]. To improve the timeliness of reporting, dedicated human resources, computers, and Internet connectivity should be made available at all sites. At the same time, it is important to ensure that the gains made in other aspects of data quality are consolidated and sustained. Efforts such as training, continuous monitoring, and supportive supervision should be continued. Future research should assess if these improvements are reflected at other surveillance sites in Nepal, as this will provide a more complete picture.

## 5. Conclusions

This OR study found significant improvements in the quality of the AMR surveillance reports submitted to the NPHL for GLASS reporting by the sites in Bagmati Province, Nepal compared to an OR study conducted in 2019. The timeliness of the submission of reports can be further improved. The AMR surveillance program in Nepal needs to sustain efforts such as training and continued supervision that are designed to improve surveillance. Focus on the provision of dedicated manpower and information technology infrastructure is needed to further improve the quality of reporting. Similar assessments should also be conducted covering surveillance sites in all provinces.

## Figures and Tables

**Table 1:** Completeness of the AMR surveillance data from AMR surveillance sites in Bagmati Province, Nepal during July–December 2022 compared to January–June 2019 ^1^.

	First OR Study January–June 2019			Second OR Study July–December 2022			*p*-Value ^3^
	Records Examined	Records Complete in All Fields ^2^		Records Examined	Records Complete in All Fields ^2^		
	N	n	%	N	n	%	
Site A	580	536	(92%)	631	631	(100%)	<0.001
Site B	3164	0	(0%)	2100	2100	(100%)	<0.001
Site C	810	0	(0%)	903	903	(100%)	<0.001
Site D	265	122	(46%)	107	107	(100%)	<0.001
Site E	341	341	(100%)	219	219	(100%)	NA
Site F ^4^	NA	NA	NA	1129	1129	(100%)	NA
Site G ^4^	NA	NA	NA	213	213	(100%)	NA
Overall	5160	999	(19%)	5302	5302	(100%)	<0.001

^1^ Data for the period January–June 2019 has been published and can be accessed at https://doi.org/10.3390/tropicalmed6020060 (accessed on 1 August 2023). ^2^ Completeness in the following fields were examined in all records: age, gender, origin, date of specimen collection, specimen, isolate, antibacterial susceptibility results. ^3^ Two-proportion Z-test. ^4^ Sites F and G had not submitted any data during January–June 2019. Abbreviations: AMR: antimicrobial resistance; NA: not applicable; OR: operational research.

**Table 2:** Timeliness of the submission of AMR surveillance reports from five AMR surveillance sites in Bagmati Province, Nepal to the National Public Health Laboratory during July–December 2022 compared to January–June 2019 ^1^.

Sites	First OR Study January–June 2019			Second OR Study July–December 2022		
	Expected Reports (N)	Timely Reports (n)	Delay in Submission Median Days (IQR)	Expected Reports (N)	Timely Reports (n)	Delay in Submission Median Days (IQR)
Site A	6	4	0 (0–17)	6	3	2 (0–4)
Site B	6	0	246 (207–284)	6	2	19 (2–36)
Site C	6	6	0 (0)	6	2	35 (5–73)
Site D	6	0	272 (233–310)	6	1	51 (13–89)
Site E	6	6	0 (0–0)	6	6	0 (0–0)
Site F ^2^	6	0	-	6	2	31 (6–51)
Site G ^2^	6	0	-	6	1	27 (3–65)

^1^ Data for the period January–June 2019 has been published and can be accessed at https://doi.org/10.3390/tropicalmed6020060 (accessed on 1 August 2023). ^2^ Sites F and G had not submitted any data during January–June 2019. Abbreviations: AMR: antimicrobial resistance, IQR: inter-quartile range, OR: operational research.

**Table 3:** Consistency in the specimen–pathogen combination according to GLASS from the reporting AMR surveillance sites in Bagmati Province, Nepal, during July–December 2022 compared to January–June 2019 ^1^.

		First OR Study January–June 2019			Second OR Study July–December 2022			
Specimen Type	Site	Total Records	Consistent		Total Records	Consistent		*p*-Value ^2^
		N	n	(%)	N	n	(%)	
Blood	Overall	403	403	(100%)	904	707	(78%)	<0.001
	Site A	47	47	(100%)	23	23	(100%)	NA
	Site B	108	108	(100%)	459	349	(76%)	<0.001
	Site C	134	134	(100%)	171	171	(100%)	NA
	Site D	99	99	(100%)	21	21	(100%)	NA
	Site E	15	15	(100%)	4	1	(25%)	<0.001
	Site F ^3^	-	-	-	205	123	(60%)	NA
	Site G ^3^	-	-	-	21	19	(90%)	NA
Urine	Overall	1147	1137	(99%)	3617	3617	(100%)	<0.001
	Site A	380	380	(100%)	494	494	(100%)	NA
	Site B	262	258	(99%)	1421	1421	(100%)	<0.001
	Site C	238	236	(99%)	633	633	(100%)	0.02
	Site D	111	107	(96%)	51	51	(100%)	0.16
	Site E	156	156	(100%)	184	184	(100%)	NA
	Site F ^3^	-	-	-	715	715	(100%)	NA
	Site G ^3^	-	-	-	119	119	(100%)	NA
Feces	Overall	27	24	(90%)	17	17	(100%)	0.15
	Site A	0	NA	NA	1	1	(100%)	NA
	Site B	8	7	(89%)	11	11	(100%)	0.22
	Site C	0	NA	NA	0	NA	NA	NA
	Site D	19	17	(91%)	3	3	(100%)	0.55
	Site E	0	NA	NA	2	2	(100%)	NA
	Site F ^3^	-	-	-	0	NA	NA	NA
	Site G ^3^	-	-	-	0	NA	NA	NA
Genital swab	Overall	4	3	(80%)	213	188	(88%)	0.41
	Site A	1	1	(100%)	1	1	(100%)	NA
	Site B	2	1	(50%)	44	41	(93%)	0.03
	Site C	0	NA	NA	99	99	(100%)	NA
	Site D	2	2	(100%)	0	NA	NA	NA
	Site E	0	NA	NA	0	NA	NA	NA
	Site F ^3^	-	-	-	67	45	(67%)	NA
	Site G ^3^	-	-	-	2	2	(100%)	NA

^1^ Data for the period January–June 2019 has been published and can be accessed at https://doi.org/10.3390/tropicalmed6020060 (accessed on 1 August 2023). ^2^ Two-proportion Z-test. ^3^ Sites F and G had not submitted any data during January–June 2019. Abbreviations: GLASS—Global Antimicrobial Resistance and Use Surveillance System; AMR—antimicrobial resistance; NA—not applicable; OR: operational research. Orange color denotes a decline in consistency; Green color denotes an improvement in consistency.

**Table 4:** Current status of the availability of basic infrastructure and specific requirements at four AMR surveillance sites in Bagmati Province, Nepal that were considered as “non-reporting” sites in 2019.

Requirements	Site F		Site G		Site H		Site I	
	2019–2020 ^1^	2022	2019–2020 ^1^	2022	2019–2020 ^1^	2022	2019–2020 ^1^	2022
Number of microbiology staff	8	7	7	4	15	14	4	3
Adequate space for data entry	Yes	Yes	None	Yes	Yes	Yes	None	None
Availability of computer with internet connectivity for data entry	No	Yes	Yes	Yes	Yes	Yes	No	No
Availability of person for data entry	No	Yes	Yes	Yes	Yes	Yes	No	No
Year of recent AMR surveillance training	2019	2021	NA	2021	2019	2021	2019	2021
Year of recent data management/WHONET training	2019	2022	NA	2022	Yearly	2022	2019	2022
Agreement on data sharing with the NPHL ^2^	Verbal	Written	Verbal	Written	Verbal	Written (signed in October 2022)	Verbal	Written (signed in August 2022)

^1^ Data for the period 2019–2020 has been published and can be accessed at https://doi.org/10.3390/tropicalmed6020060 (accessed on 1 August 2023). ^2^ Verbal indicates the presence of an informal understanding regarding AMR data sharing while written indicates the presence of a formal memorandum of understanding between the site and the National Public Health Laboratory regarding AMR data sharing. Abbreviations: AMR—antimicrobial resistance, NA—not applicable, WHONET—WHO free software, NPHL—National Public Health Laboratory.

## Data Availability

The data contain sensitive information that were obtained from various AMR surveillance sites after approval from the relevant authorities and in-country ethics committees. We have permission to share only aggregate, analyzed data but not individual patient-wise data. Therefore, the data cannot be made publicly available. However, if anyone provides a justifiable reason to access the individual patient-wise de-identified data, they are asked to contact the corresponding author (upadhaya.sweety@gmail.com) or the National Public Health of Nepal. Email: sweety@nphl.gov.np (institutional email address). Mailing address: National Public Health Laboratory, Department of Health Services, Ministry of Health and Population, Tripura Marg, Teku, Kathmandu, Nepal. Postal code 44600. Tel.: +977-9847551518; +977-01-5352421.

## Supplementary Materials

The following supporting information can be downloaded at: https://www.mdpi.com/article/10.3390/tropicalmed8080399/s1, The following is available online at Table S1. Priority specimens and pathogens for surveillance of AMR according to GLASS criteria for Nepal. Table S2. Pathogen–antibacterial combinations on which GLASS gathers data; Table S3. Operational definitions; Table S4. Consistency in pathogen–antibacterial combination of AMR surveillance data according to GLASS from five AMR surveillance sites in Bagmati Province, Nepal during July–December 2022 compared to January–June 2019.

## Open Access Statement

In accordance with the WHO’s open-access publication policy for all work funded by the WHO or authored/co-authored by WHO staff members, the WHO retains the copyright of this publication through a Creative Commons Attribution IGO license (http://creativecommons.org/licenses/by/3.0/igo/legalcode accessed on 1 August 2023), which permits unrestricted use, distribution, and reproduction in any medium provided the original work is properly cited.
