# Supplementary material for: Has Data Quality of an Antimicrobial Resistance Surveillance System in a Province of Nepal Improved between 2019 and 2022?
Source: Trop Med Infect Dis. 2023 Aug 4;8(8):399. doi: 10.3390/tropicalmed8080399 (PMC10459402; doi:10.3390/tropicalmed8080399)
Supplement: Supplementary file 1 [file tropicalmed-08-00399-s001.zip › tropicalmed-2496848-supplementary.pdf]

Table S1. Priority specimens and pathogens for surveillance of AMR[1] according to GLASS[2] criteria for Nepal

Specimen	Priority pathogens for surveillance
	Escherichia coli
Blood	*Klebsiella pneumoniae*<br>*Acinetobacter baumannii*<br>*Staphylococcus aureus*<br>*Streptococcus pneumoniae*<br>*Salmonella* spp.[3]
Urine	*Escherichia coli*<br>*Klebsiella pneumoniae*
Stool	*Salmonella* spp.<br>*Shigella* spp.
Genital swabs	*Neisseria gonorrhoeae*

[1]AMR, antimicrobial resistance; [2]GLASS, Global Antimicrobial Resistance Surveillance System;[3]spp., species

Table S2. Pathogen–antibacterial combinations on which GLASS[1] gathers data

Pathogen	Antibacterial agents that may be used for AST[2]
Escherichia coli	Ampicillin<br>Co-trimoxazole<br>Ciprofloxacin or levofloxacin<br>Ceftriaxone or cefotaxime and ceftazidime<br>Cefepime<br>Imipenem, meropenem, ertapenem, or doripenem<br>Colistin
Klebsiella pneumoniae	Co-trimoxazole<br>Ciprofloxacin or levofloxacin<br>Ceftriaxone or cefotaxime and ceftazidime,<br>Cefepime<br>Imipenem, meropenem, ertapenem, or doripenem<br>Colistin
Acinetobacter spp.[3]	Tigecycline or minocycline<br>Gentamicin and amikacin<br>Imipenem, meropenem, or doripenem<br>Colistin
Streptococcus pneumoniae	Penicillin G<br>Co-trimoxazole Ceftriaxone or cefotaxime<br>Ciprofloxacin or Levofloxacin
Salmonella spp.[3]	Ceftriaxone or Cefotaxime and Ceftazidime<br>Imipenem, Meropenem, Ertapenem, or Doripenem
Shigella spp.[3]	Ciprofloxacin or levofloxacin<br>Ceftriaxone or cefotaxime and ceftazidime<br>Azithromycin
Neisseria gonorrhoeae	Cefixime<br>Ceftriaxone<br>Azithromycin<br>Spectinomycin<br>Ciprofloxacin<br>Gentamicin

[1]GLASS, Global Antimicrobial Resistance Surveillance System; [2]AST: antibiotic susceptibility test- ing; [3]spp., species

Table S3. Operational definitions

Terminiology	Operational definition
AMR data	AMR data is defined as the detailed identification and antibiotic susceptibility report along with unique identifiers, specimen, origin, date of sampling and demographic data from surveillance sites' microbiology laboratory records.
Deadline for report submission to NPHL	For a particular month, the deadline for submission of surveillance reports by the surveillance site to NPHL is the last working day of the following month.
Timeliness of report submission	Report of AMR surveillance, for a particular month, received within deadline for submission, i.e., last working day of the following month.
Specimen-pathogen combination	Combination of priority specimens (namely, blood, urine, stool, or genital swabs) with priority pathogens according to the GLASS. The valid combinations of specimen-pathogen as per GLASS are provided in Table S1.
Pathogen-antibacterial combination	Combination of eight priority pathogens and the relevant listed antibiotics according to the GLASS. The valid combinations of pathogen-antimicrobial as per GLASS are provided in Table S2.
Completeness of data	Completeness signifies no missing variables required according to GLASS criteria: age, sex, pathogen, origin, specimen, antibiotic susceptibility results and date of specimen collection.
Non-reporting sites	The surveillance sites that have not sent any AMR laboratory data to NPHL for 90 consecutive days will be defined as "non-reporting" sites.
Basic infrastructure	The definition of basic infrastructure is the basic facilities and equipment required by the AMR surveillance site to send the AMR reports to NPHL.
Specific requirements	Includes the specific requirements such as qualification of data entry person, trainings received on AMR surveillance, data management, WHONET, and Agreement with NPHL/TOR, institutional restrictions on data sharing (other than the basic infrastructure) to send the AMR reports regularly to NPHL.

Abbreviations: AMR: Antimicrobial resistance, GLASS: Global Antimicrobial Surveillance and Use System, NPHL: National Public Health Laboratory

Table S4. Consistency in pathogen-antibacterial combination of AMR surveillance data according to GLASS from five AMR surveillance sites in Bagmati Province of Nepal during July-December 2022 compared to January-June 2019[1]

Pathogens	First OR study January-June 2019			Second OR study July-December 2022			p-value[2]
	Total records examined N	Consistent records n	(%)	Total records examined N	Consistent records n	(%)	
**Overall**							
Escherichia coli	1020	641	(63)	2571	2147	(84)	<0.001
Klebsiella pneumoniae	242	127	(52)	565	435	(77)	<0.001
Acinetobacter baumanii	64	17	(27)	173	173	(100)	<0.001
Staphylococcus aureus	168	73	(43)	60	44	(73)	<0.001
Streptococcus pneumoniae	1	0	(0)	8	2	(25)	NA
Salmonella species	251	153	(61)	183	179	(98)	<0.001
Shigella species	103	12	(12)	2	2	(100)	0.0003
Neisseria gonorrhoeae	4	0	(0)	5	4	(80)	0.0020
**Site A**							
Escherichia coli	354	172	(49)	361	315	(87)	<0.001
Klebsiella pneumoniae	31	15	(48)	44	38	(86)	0.0004
Acinetobacter baumanii	4	4	(100)	0	0	NA	NA
Staphylococcus aureus	4	0	(0)	2	2	(100)	0.01
Streptococcus pneumoniae	0	NA	NA	0	0	NA	NA
Salmonella species	34	32	(94)	13	11	(85)	0.29
Shigella species	0	NA	NA	0	0	NA	NA
Neisseria gonorrhoeae	1	0	(0)	0	0	NA	NA
**Site B**							
Escherichia coli	220	110	(50)	1134	1017	(90)	<0.001
Klebsiella pneumoniae	67	37	(55)	261	221	(85)	<0.001
Acinetobacter baumanii	15	0	(0)	21	21	(100)	<0.001
Staphylococcus aureus	19	19	(100)	16	16	(100)	NA
Streptococcus pneumoniae	0	NA	NA	8	2	(25)	NA
Salmonella species	56	55	(98)	152	151	(99)	0.45
Shigella species	3	0	(0)	1	1	(100)	0.045
Neisseria gonorrhoeae	1	0	(0)	5	4	(80)	NA
**Site C**							
Escherichia coli	218	199	(91)	422	201	(48)	<0.001
Klebsiella pneumoniae	94	44	(47)	114	35	(31)	0.017
Acinetobacter baumanii	44	13	(30)	96	96	(100)	<0.001
Staphylococcus aureus	16	16	(100)	0	0	NA	NA
Streptococcus pneumoniae	0	NA	NA	0	0	NA	NA
Salmonella species	0	NA	NA	0	0	NA	NA
Shigella species	0	NA	NA	0	0	NA	NA
Neisseria gonorrhoeae	0	NA	NA	0	0	NA	NA
**Site D**							
Escherichia coli	109	93	(85)	34	15	(44)	<0.001
Klebsiella pneumoniae	10	7	(70)	5	5	(100)	0.17
Acinetobacter baumanii	1	0	(0)	4	4	(100)	NA
Staphylococcus aureus	29	29	(100)	8	8	(100)	NA

Streptococcus pneumoniae	1	0	(0)	0	0	NA	NA
Salmonella species	66	63	(95)	13	12	(92)	0.63
Shigella species	14	12	(86)	0	0	NA	NA
Neisseria gonorrhoeae	2	0	(0)	0	0	NA	NA
**Site E**							
Escherichia coli	119	67	(56)	116	114	(98)	<0.001
Klebsiella pneumoniae	40	24	(60)	22	20	(91)	0.01
Acinetobacter baumanii	0	NA	NA	0	0	NA	NA
Staphylococcus aureus	100	9	(9)	4	3	(75)	0.001
Streptococcus pneumoniae	0	0	NA	0	0	NA	NA
Salmonella species	95	3	(3)	1	1	(100)	<0.001
Shigella species	86	0	(0)	1	1	(100)	<0.001
Neisseria gonorrhoeae	0	NA	NA	0	NA	NA	NA
**Site F[3]**							
Escherichia coli	-	-	-	411	400	(97)	NA
Klebsiella pneumoniae	-	-	-	109	106	(97)	NA
Acinetobacter baumanii	-	-	-	47	47	(100)	NA
Staphylococcus aureus	-	-	-	23	12	(52)	NA
Streptococcus pneumoniae	-	-	-	0	NA	NA	NA
Salmonella species	-	-	-	0	NA	NA	NA
Shigella species	-	-	-	0	NA	NA	NA
Neisseria gonorrhoeae	-	-	-	0	NA	NA	NA
**Site G[3]**							
Escherichia coli	-	-	-	93	85	(91)	NA
Klebsiella pneumoniae	-	-	-	10	10	(100)	NA
Acinetobacter baumanii	-	-	-	5	5	(100)	NA
Staphylococcus aureus	-	-	-	7	3	(43)	NA
Streptococcus pneumoniae	-	-	-	0	NA	NA	NA
Salmonella species	-	-	-	4	4	(100)	NA
Shigella species	-	-	-	0	NA	NA	NA
Neisseria gonorrhoeae	-	-	-	0	NA	NA	NA

[1]Data for period January-June 2019 has been published and can be accessed at 10.3390/tropicalmed6020060 (last accessed on 01 Aug 2023)
[2] Two-proportion Z-test
[3]Sites F and G had not submitted any data during January-June 2019
Abbreviations: AMR: Antimicrobial resistance, GLASS: Global Antimicrobial Resistance Surveillance System, NA: Not applicable, OR: Operational Research
Pink Color denotes a decline in consistency; Green Color denotes an improvement in consistency
